# A survey of mental health literacy in parents and guardians of teenagers

**DOI:** 10.3389/fpsyt.2024.1295918

**Published:** 2024-02-09

**Authors:** Sakurako Kusaka, Jerome Clifford Foo, Satoshi Yamaguchi, Ayuko Yukawa, Takuya Arai, Fumika Sawamura, Fumiharu Togo, Tsukasa Sasaki

**Affiliations:** ^1^ Department of Physical and Health Education, Graduate School of Education, The University of Tokyo, Tokyo, Japan; ^2^ Research Fellow of Japan Society for the Promotion of Science, Tokyo, Japan; ^3^ Institute for Psychopharmacology, Medical Faculty Mannheim, Central Institute of Mental Health, University of Heidelberg, Mannheim, Germany; ^4^ Department of Genetic Epidemiology in Psychiatry, Medical Faculty Mannheim, Central Institute of Mental Health, University of Heidelberg, Mannheim, Germany; ^5^ Department of Psychiatry, College of Health Sciences, University of Alberta, Edmonton, AB, Canada; ^6^ Unit for Mental Health Promotion, Research Center for Social Science & Medicine, Tokyo Metropolitan Institute of Medical Science, Tokyo, Japan; ^7^ Saitama Prefectural Education Bureau Student Consultation Division, Saitama, Japan; ^8^ Saitama Prefectural Education Bureau Health and Physical Education Division, Saitama, Japan

**Keywords:** mental health literacy, parents, caregivers, teenagers, health education

## Abstract

**Introduction:**

Parents and guardians (hereafter caregivers) of teenagers need high levels of mental health literacy (MHL) to manage mental health problems arising in teenagers in their care. Previous studies assessing MHL levels in caregivers of teenagers have reported mixed results, making it difficult to clearly estimate caregiver MHL levels. This study aimed to investigate MHL levels in Japanese caregivers of regular teenagers.

**Methods:**

Responses from caregivers (n = 1,397) of students entering junior and senior high schools to a self-administered online questionnaire were analyzed. The questionnaire assessed (a) knowledge about mental health/illnesses and (b) attitudes towards mental health problems in teens in their care (e.g., recognition of depression as a medical illness and intention to engage in helping behaviors).

**Results:**

The average proportion of correct answers to the knowledge questions (n = 7) was 55.4%; about one tenth (9.2%) of caregivers correctly answered only one or none of the questions. Few caregivers correctly answered about the life-time prevalence of any mental illnesses (46.1%) and appropriate sleep duration for teenagers’ health (16.5%). The proportions of caregivers who had the intention to listen to the teen in their care, consult another person, and seek professional medical help if the teen suffered from depression were 99.5%, 91.5% and 72.7%, respectively.

**Conclusions:**

Many teenagers’ caregivers appeared to be willing to help the teens in their care if they were suffering from mental health problems. However, there was much room for improvement in knowledge on mental health/illnesses and intention to seek help from medical professionals. Efforts toward better education should be made.

## 1 Introduction

The prevalence of mental illnesses sharply increases as children reach their teenage years (1). However, teenagers may have difficulty in recognizing their own mental health (MH) problems (2), and even when they can, they may be reluctant to seek help (3). Therefore, teenagers may need the help of parents or other supportive adults so that they can recognize their own problems and seek appropriate help (2).

Given that parents and/or guardians (hereafter caregivers) are the most familiar adults for most teenagers, caregivers need to be aware of MH problems in the teens in their care and appropriately manage these problems; this requires adequate mental health literacy (MHL). MHL is defined as knowledge and beliefs which aid in the recognition, management or prevention of MH problems (4). More specifically, knowledge about mental health/illnesses, recognition of mental disorders, and attitudes towards appropriate help-seeking are examples of components of MHL (2, 4).

Even caregivers of teens without MH problems need MHL because they need to be able to recognize and manage problems which can occur in future. Levels of MHL in caregivers of regular teens have been studied in a number of previous studies (5–17). The most studied aspect of MHL was knowledge about mental health/illness, with high variation in knowledge levels observed between studies (5, 6, 12, 14, 15). Other components of MHL were also examined including intention to help their teens (10, 11, 13), and belief that a teen with MH problems is weak, not ill (12). In addition, most of the previous studies did not clearly report response rates, leaving room for bias (5–7, 12–17). These mixed results make the estimation of MHL in caregivers of teenagers challenging.

The present school-based study aimed to examine MHL levels in caregivers of regular teenagers. The survey was conducted at school entrance ceremonies, which almost all caregivers, not only those who were interested in MH, attended; this was done to reduce selection bias as much as possible. Analyses were conducted in the whole sample, as well as stratified by age and gender given significant age and gender-related differences in MHL previously observed in the general adult population (18–20).

## 2 Methods

### 2.1 Participants and procedure

A call was put out through the Prefectural board of education in Saitama, Japan, for public junior and senior high schools to participate in various studies on MH education for students and caregivers. The goal was to recruit 8 junior and 8 senior high schools. Twenty-seven junior and 5 senior high schools applied; 8 junior high schools were randomly selected from the 27, and all the 5 senior high schools were included. Of these, 3 junior and 4 senior high schools participated in the MH program involving the present research in 2022. The 7 participating schools are located in suburban metropolitan areas. In Japan, the majority of junior/senior high school students attend public schools. Unlike public junior high schools, public senior high schools have entrance examinations and the achievement levels of the 4 participating high schools on these exams were within ± 1 SD of the national average. The survey was conducted at the school entrance ceremony, which most caregivers of the new students attended. The second half of the ceremony included a MH seminar. Before the MH seminar started, caregivers who attended the ceremony were given a QR code and asked to answer a self-administered online questionnaire about MHL on their own mobile devices. Of 1,741 new students, 1,820 caregivers attended the entrance ceremony (multiple people, e.g. both parents, participated in some families), and of those, 1,655 answered the questionnaire. Of those, 1,562 agreed to have their responses analyzed and participate in this study, completing the online submission of informed consent (response rate = 85.8%). A further n=131 who were found to have submitted their responses after start of the MH seminar were excluded from the analysis. Respondents whose age was under 30 (n = 23) or whose mother tongue was not Japanese (n = 11) were also excluded. In the end, responses from 1,397 caregivers were analyzed. The study was approved by The University of Tokyo Human Research Ethics Committee (#21-348).

### 2.2 Assessments

#### 2.2.1 Demographic variables

In the first part of the questionnaire, the demographics of caregivers including age, gender, having/previously having a job related to MH, and previous participation in MH seminars were assessed (**Table 1**).

**Table 1:** Demographic data of caregivers.

Characteristics	Number (%)
**Total**	1397
Age	
30’s	200 (14.3%)
40’s	958 (68.6%)
50’s	235 (16.8%)
60’s	3 (0.2%)
70’s and over	1 (0.1%)
Gender	
Male	213 (15.2%)
Female	1178 (84.3%)
No answer	6 (0.4%)
Previous participation in MH seminars	
None	1280 (91.6%)
Yes	117 (8.4%)
Having/previously having a job related to MH	
None	1317 (94.3%)
Yes	80 (5.7%)

#### 2.2.2 Knowledge about mental health/illnesses (knowledge)

In the second part, basic knowledge about mental health/illnesses was assessed using seven questions (see **Table 2**). The questions were developed by the authors (a team including psychiatrists, school teachers, and school nurses). Topics were chosen to determine the extent to which caregivers understood the importance of attention to, care of and prevention of mental illness and suicide risk in teenagers. Questions related to the increase in the prevalence of mental illness (21) and suicide (22) among teenagers (suicide is the leading cause of death among older teens in Japan (23)) and the impact of mental illness on teens’ lives including their academic achievement, were included. Questions about the amount of sleep needed to maintain MH were included, since shorter sleep durations have been associated with increased risk of mental illness and suicidality (24–27), and many teenagers worldwide are found to sleep shorter (28) than the recommended sleep duration for their age (29). Three of the seven questions were modified from items used in a previous study by the authors (30). The questions were to be answered, “True”, “False”, or “I don’t know”. The internal consistency (Cronbach’s alpha) of the 7 questions was 0.56 in the present sample.

**Table 2:** Correct responses to each of the knowledge questions about mental health/illnesses.

Items	Correct answer	Proportion of correct responses (%)
About one in five people will experience a mental illness in their lifetime.	T	46.1
The incidence of mental illnesses starts to sharply increase in the early teens.	T	73.9
Children who study hard and are more active in their extracurricular activities are less likely to suffer from mental illnesses.	F	55.1
Most children will not talk to others about their mental health problems on their own.	T	82.7
Mental health problems are unrelated to academic achievement.	F	56.8
Suicide is the most common cause of death among older teens in Japan.	T	56.5
7 hours per night is the best amount of sleep for health in junior and senior high school students.	F	16.5
The average number of correct responses to the 7 questions		3.9/7 (55.4%)

T, True; F, False.

#### 2.2.3 Attitudes towards mental health problems

In the third part, caregivers were asked to read a case vignette describing a teen with symptoms of major depression (see below), and to imagine that the teen in their care is in the same situation as Teen A. The vignette was written by the authors, with reference to the one in Jorm et al. (2007) (10) and the criterion A of DSM-5 criteria of major depressive disorder (31).

##### Vignette

Teen A has often been late for school over the last 2 weeks. They feel tired and can’t keep their mind on classes. Teen A says that they have trouble sleeping, don’t feel like eating, and don’t have fun watching their favorite TV program. Studying also seems to be difficult for them.

Then, caregivers were asked to choose responses to the following questions which matched their thoughts most closely (2.2.3.1, 2.2.3.2, 2.2.3.3).

##### 2.2.3.1 Recognition of depression as medical illness

Caregivers were asked to what extent they agreed with the following (3 out of the 4) “weak-not-sick” items of the Depression Stigma Scale (32): 1) “The teen could snap out of it if they wanted”; 2) “The teen’s problem is a sign of personal weakness”; 3) “The teen’s problem is not a real medical illness”. The fourth item, “It is best to avoid people with a problem like this child so that you do not develop this problem” was not asked because the item was not relevant to the recognition of depression in teens under the caregivers’ care. Possible responses to each item were as follows: “Agree”, “Somewhat Agree”, “Somewhat Disagree”, or “Disagree”. “Somewhat Disagree” and “Disagree” were considered the desired responses.

##### 2.2.3.2 Intention to engage in helping behaviors if the teen suffers from depression

Caregivers were asked “What would you do in this situation?” to assess the intention to help if the teen in their care were in the same mental state as Teen A. Specifically, they were asked to answer whether they would: 1) listen to what the teen has to say; 2) consult someone about the teen; and 3) seek medical help. Possible responses to each item were “Yes”, “Probably yes”, “Probably no”, or “No”. Caregivers were considered to have the intention to engage in the behavior, when the answer was “Yes” or “Probably yes” (“Yes” and “Probably yes” were considered the desired responses).

##### 2.2.3.3 Acceptance of the teen’s diagnosis

Caregivers were asked “If the teen in your care were diagnosed with a mental illness, how would you feel about the diagnosis?” Possible responses to the question were “I would be able to accept the diagnosis”, “I would probably be able to accept the diagnosis”, “I would probably not be able to accept the diagnosis”, or “I would not be able to accept the diagnosis”. Caregivers were considered to be able to accept the diagnosis, when the response was “I would be able to accept the diagnosis” or “I would probably be able to accept the diagnosis”.

### 2.3 Statistical analysis

Proportions of correct responses to the knowledge questions and desirable responses to other questions were summarized in all subjects, as well as by age and gender. To examine differences by age/gender, linear or binary logistic regression were performed employing the number of correct responses or whether caregivers had desirable responses as dependent variables, adjusting for experience of having a job related to MH, and previous participation in MH seminar.

## 3 Results

### 3.1 Demographic variables

**Table 1** shows demographic characteristics of the participants (n = 1,397). Most (84.3%, n = 1,178) of the participants were female, and two-thirds (68.6%, n = 958) were in their 40’s. The proportion of caregivers who had participated in other MH seminars was 8.4% (n =117), and who had had a job related to MH was 5.7% (n = 80).

### 3.2 Knowledge about mental health/illnesses

**Figure 1:**
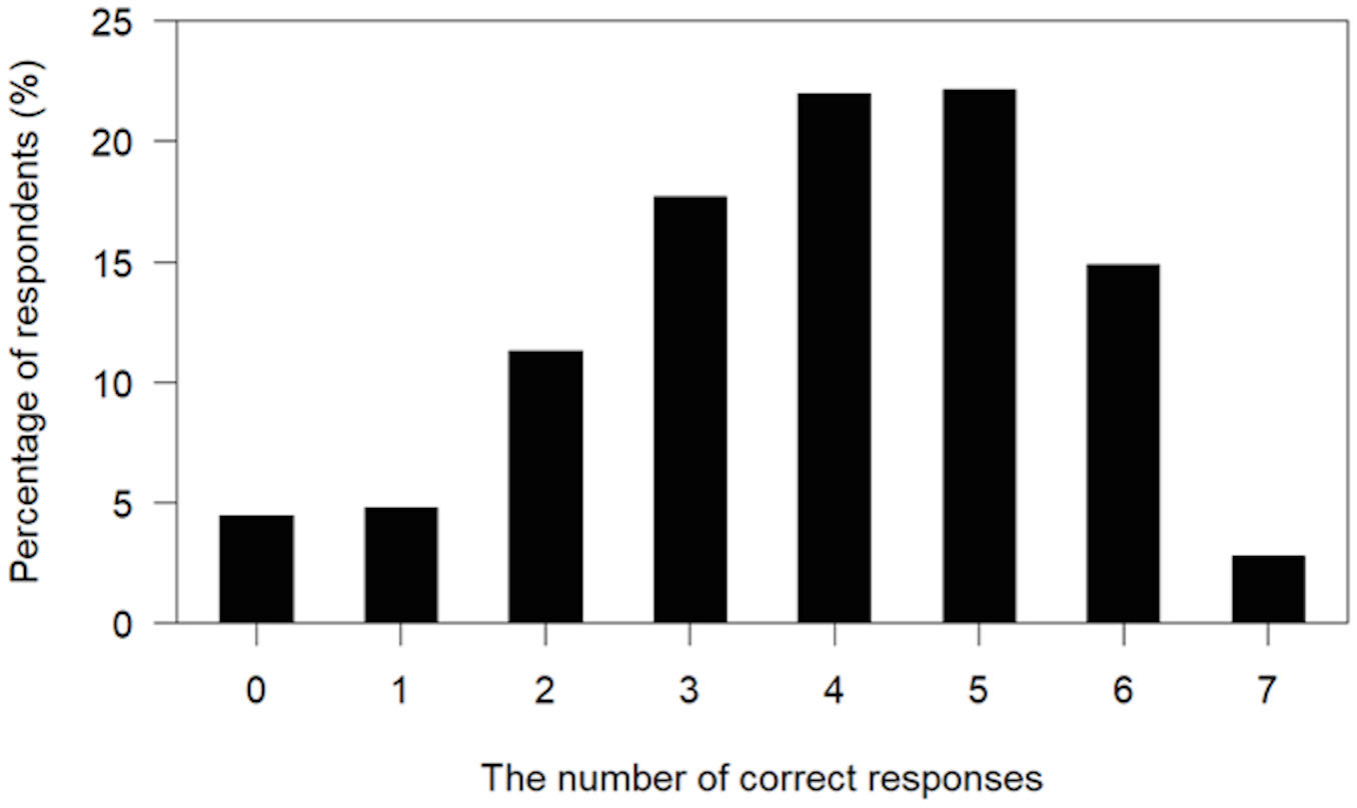
Distribution of the number of correct responses in caregivers to the 7 knowledge questions about mental health/illnesses.

**Figure 1** shows the distribution of the number of correct responses to the knowledge questions in the caregivers. The average number of the correct responses was 3.9 (out of 7 questions; standard deviation = 1.7). About one tenth (9.2%) of caregivers correctly answered only one or none of the questions; 38.2% correctly answered to less than half (3 or less) of the questions. **Table 2** summarizes the proportion of correct responses to each question. The proportion was low (around or below half) in the following questions: appropriate sleep duration for adolescents at the age of junior and senior high school (16.5%), life-time prevalence of any mental illnesses (46.1%), MH in students who study hard (55.1%), relationship of MH and academic achievement (56.8%), and suicide being the leading cause of death in late teens (56.5%).

### 3.3 Attitudes towards mental health problems

**Table 3** summarizes responses to questions about recognition of depression as a medical illness and about intention to help teens, when teens in their care were assumed to be in the same situation as Teen A in the vignette. The majority of caregivers disagreed/somewhat disagreed that the problem was not a real medical illness, that the problem was a sign of personal weakness, and that the teen could snap out of it if they wanted. Regarding the intention to help, most caregivers answered that they would/would probably listen to what the teen has to say, consult someone about the teen, and seek medical help. Most caregivers (95.4%) answered that they would/would probably accept having teens in their care diagnosed with a mental illness (not shown in tables).

**Table 3:** Responses to questions about recognition of depression as a medical illness, intention to help, and acceptance of the teen’s diagnosis.

Items	Proportion of responses (%)
Desirable responses to “recognition of depression as medical illness”	Disagree/Somewhat disagree
The teen could snap out of it if they wanted.	73.5
The teen’s problem is a sign of personal weakness.	84.5
The teen’s problem is not a real medical illness.	88.9
Desirable responses to “intentions to help teens”	Yes/Probably yes
I will listen to what the teen has to say.	99.5
I will consult someone about the teen.	91.5
I will seek medical help.	72.7
If the teen in your care were diagnosed with a mental illness, how would you feel about the diagnosis?	Would be able to accept/Would probably be able to accept
	95.4

### 3.4 MHL in caregivers by gender and age

Proportions of correct/desired responses by gender and age are summarized in **Supplementary Table 1**. No difference by age was observed in any questions/items. By gender, the proportions appear to be higher in females than males in the items including “The teen’s problem is a sign of personal weakness”, “I will consult someone about the teen”, and “I will seek medical help”; the differences were statistically significant (odds ratio = 2.5, 4.0 and 2.2, respectively, all p < 0.001, reference = males) adjusting for age, current/previous experiences of job related to MH, and previous participation in MH seminar.

## 4 Discussion

The present study examined the level of MHL among caregivers of regular teenagers. Most caregivers had the desired attitudes towards potential MH problems in teens in their care, except for when it came to seeking professional help. In contrast, knowledge about MH was not sufficient in most caregivers.

Caregivers appear prepared to take basic steps to help if teens in their care have MH problems and the intention to engage in helping behaviors was high; most caregivers answered that they would listen to the teens and try to consult someone about the problems. However, 30% of caregivers did not consider seeking professional help for potential symptoms of depression. This appears in line with previous research, which found that 30% of caregivers would not seek help from doctors (e.g., general practitioners) when teens in their care suffered from depressive symptoms (11). One possible reason contributing to this could be the young people’s unwillingness to visit doctors (11). Based on the present study, emotional unacceptance of the diagnosis may not be a major barrier; most of the present caregivers answered that they could accept having teens in their care diagnosed with a mental illness. It does not appear that not recognizing of depression and its symptoms as a medical illness was a major barrier to seeking treatment; most of the present caregivers recognized the teen in the vignette to be suffering from a medical illness. The reason why caregivers do not seek professional help for the teens in their care needs to be further studied.

Caregivers’ knowledge on mental health/illnesses was insufficient. A substantial portion (10%) of the caregivers correctly answered only one or zero questions; one third correctly answered less than half of the questions. Specifically, half of the caregivers did not know that suicide is the leading cause of death among older Japanese teenagers, that lifetime prevalence of mental illnesses reaches 20% in Japan, and that MH problems are related to academic achievement. Most of the caregivers did not know that the appropriate amount of sleep for teenagers’ health is 8-10 hours (29). In previous studies, while the percentage of correct answers was high (> 75%) in studies asking questions about risk factors, symptoms/influences and treatment of MH problems (6, 15), percentages were lower in a study asking questions such as how to support teenagers with the problems (55%) (12). Another study reported that only half of caregivers knew that medication can be an effective treatment for MH problems and that a full recovery was possible (14). Improving caregivers’ knowledge about appropriate support for teens and lifestyles such as sleep amount is needed. While knowledge can play a role in predicting health behaviors, alone it cannot predict or change behavior; on the other hand it is suggested that beliefs (attitude or intention) related to health may be key predictors (33). For caregivers to take action to encourage a teen to access MH services can involve steps such as recognizing the problem as psychological in nature, considering possible courses of action, and developing the intention to seek out MH services for the teen (34). Knowledge can inform each of these steps, and how it does in real world situations requires further study.

We did not observe effects of age or gender on knowledge. However, there are many other potential confounding factors on the pathway from improved MHL to improved care and outcomes for youth. The diversity of mental health presentations in teenagers, which can make mental illnesses challenging to recognize (30), stigma (including caregiver stigma in accessing MH services), as well as systemic-structural aspects of MH services influencing availability and accessibility (35), can affect access to MH care. Also, given that schools and outpatient settings are the most common locations for youth to receive MH care (36), future research should examine caregivers’ attitudes towards and behaviors of accessing school-based services. Improved in-school access to resources for well-being and welfare may help to intervene before medical services become needed.

Knowledge about mental health/illnesses did not differ by gender in caregivers, but desired attitudes toward MH problems in teens in their care were significantly more frequent in females than males. This appears in line with previous studies in regular adults; females had more desirable attitudes toward MH problems than males (18, 19). Thus far, one previous study had examined caregivers’ MHL by gender, with no significant differences observed (17). A limitation of the present results was that the proportion of males in the participants was low (15.2%), which makes it difficult to draw strong conclusions.

A strength of this study was the high response rate (85.8%). Also, caregivers were asked to answer the questionnaire during the entrance ceremony, which almost all caregivers attended. Thus, the responses are obtained from a sample of caregivers including not only respondents who were interested or knowledgeable about MH, but also those who were not, limiting bias in characteristics of participants. The present study has several limitations. First, participants were caregivers from a single prefecture in Japan. Caution may be needed when generalizing results to other populations. Studies in other regions of Japan (as well as in other countries) will be needed to obtain a more comprehensive picture of caregiver MHL. Second, we did not assess the relationship between the caregivers and teens in their care; while in Japan the majority of caregivers who attend school entrance ceremonies are parents, in future research it would be of interest to study whether MHL differs in parents and other adult caregivers. Third, to fit within time constraints, this study used a single case vignette of a teen with major depression to assess caregivers’ attitude towards mental illnesses in teens in their care. The use of a greater number of scenarios and assessment of attitudes towards other mental illnesses are needed in future studies to give a more comprehensive picture of the situation. Finally, as mentioned above, more desired attitudes toward MH problems in teens were indicated by female than male caregivers, but the proportion of males was relatively small. Future studies with more male respondents will be needed to better understand their attitudes towards mental health.

## 5 Conclusions

The present study observed that teenagers’ caregivers had good intentions to help the teens in their care if they suffered from MH problems. At the same time, it was found that caregivers have room for improvement in MHL, as they had limited knowledge about mental health/illnesses, and many did not have the intention to seek help from professionals if the teens in their care experienced potential MH symptoms. Educational programs about risk for mental illnesses and their care and prevention are needed to improve caregiver MHL.

## Data availability statement

The raw data supporting the conclusions of this article will be made available by the authors, without undue reservation.

## Ethics statement

The studies involving humans were approved by The University of Tokyo Human Research Ethics Committee. The studies were conducted in accordance with the local legislation and institutional requirements. Written informed consent for participation was not required from the participants or the participants’ legal guardians/next of kin because all participants provided electronic informed consent.

## Author contributions

SK: Conceptualization, Data curation, Formal analysis, Funding acquisition, Methodology, Writing – original draft, Writing – review & editing. JF: Writing – review & editing. SY: Methodology, Writing – review & editing. AY: Data curation, Writing – review & editing. TA: Investigation, Writing – review & editing, Methodology. FS: Investigation, Writing – review & editing, Methodology. FT: Supervision, Writing – review & editing. TS: Conceptualization, Funding acquisition, Methodology, Project administration, Supervision, Writing – review & editing.

## Supplementary material

Supplementary Table 1 Average proportion (%) by gender and age of responses to questions about MH knowledge, attitudes towards MH problems and acceptance of the teen’s diagnosis.
