# Supplementary material for: A survey of mental health literacy in parents and guardians of teenagers
Source: Front Psychiatry. 2024 Feb 9;15:1295918. doi: 10.3389/fpsyt.2024.1295918 (PMC10884296; doi:10.3389/fpsyt.2024.1295918)
Supplement: Supplementary Table 1 — Average proportion (%) by gender and age of responses to questions about MH knowledge, attitudes towards MH problems and acceptance of the teen’s diagnosis. [file Table_1.docx]

**Supplementary Table 1. Average proportion (%) by gender and age of responses to questions about MH knowledge, attitudes towards MH problems and acceptance of the teen’s diagnosis**

| **Proportion of responses (%)** | **Gender** | | **Age** | | |
| --- | --- | --- | --- | --- | --- |
|  | **Male**  **(n=213)** | **Female**  **(n=1178)** | **30’s**  **(n=200)** | **40’s**  **(n=958)** | **50’s and over**  **(n=239)** |
| Correct responses to knowledge questions | 57 | 55 | 53 | 56 | 53 |
| Desirable responses to “recognition of depression as medical illness” | | |  |  |  |
| *The teen could snap out of it if they wanted.* | 73 | 74 | 73 | 74 | 72 |
| *The teen’s problem is a sign of personal weakness.* | 73 | 87*** | 84 | 84 | 88 |
| *The teen’s problem is not a real medical illness.* | 86 | 89 | 87 | 89 | 90 |
| Desirable responses to “intention to help teens” | | |  |  |  |
| *I will listen to what the teen has to say.* | 100 | 99 | 100 | 99 | 100 |
| *I will consult someone about the teen.* | 79 | 94*** | 90 | 92 | 91 |
| *I will seek medical help.* | 58 | 75*** | 69 | 73 | 73 |
| Whether they can accept the teen’s diagnosis | 95 | 96 | 97 | 95 | 97 |

*** p < 0.001
